# Associations between hormones, metabolic markers, and bone mass in perimenopausal and postmenopausal women

**DOI:** 10.1007/s00774-025-01595-x

**Published:** 2025-03-05

**Authors:** Bingru Lu, Qunxiao Han, Shiyu Zhao, Shan Ding, Guolin Bao, Yiqing Liu

**Affiliations:** 1 Department of Clinical Laboratory, Shandong Provincial Hospital Affiliated to Shandong First Medical University, 324 Jingwuweiqi Rd, Huaiyin, Jinan 250021 Shandong, People’s Republic of China; 2 Department of Clinical Laboratory, Binzhou Central Hospital, Binzhou, 251700 Shandong Province China

**Keywords:** Postmenopausal osteoporosis, Bone mineral density, Bone mass loss, Follicle-stimulating hormone, Glucose

## Abstract

**Introduction:**

To explore the associations between hormones, metabolic markers, and low bone mass in perimenopausal and postmenopausal women.

**Materials and methods:**

A total of 198 women were enrolled in this study. The correlations between hormones, metabolic markers, and BMD were analyzed. Risk factors for bone loss were identified. Receiver operating characteristic (ROC) curves were used to display the predictive power of these risk factors.

**Results:**

The years since menopause and the levels of glucose (GLU), follicle-stimulating hormone (FSH), and luteinizing hormone (LH) were inversely correlated with BMD, while estrogen and testosterone were positively correlated with BMD. Age (odds ratio [OR] 1.232; 95% confidence interval [CI] 1.106–1.372; *p* < 0.001), GLU (OR 1.848; 95% CI 1.116–3.059; *p* = 0.017), and FSH (OR 1.089; 95% CI 1.003–1.182; *p* = 0.042) were identified as risk factors for bone loss. Age (AUC = 0.884, 95% CI 0.833–0.935), FSH (AUC = 0.824, 95% CI 0.760–0.888), and GLU (AUC = 0.683, 95% CI 0.599–0.768) demonstrated significant discrimination capability for bone loss. The combined application of these factors resulted in a better prediction effect (AUC = 0.930, 95% CI 0.893–0.967).

**Conclusions:**

Age, FSH, and GLU were found to be specific risk factors for bone loss. The utilization of these factors offers compelling predictive power for bone loss in perimenopausal and postmenopausal women.

**Supplementary Information:**

The online version contains supplementary material available at 10.1007/s00774-025-01595-x.

## Introduction

Postmenopausal osteoporosis (PMOP) is a systemic metabolic bone disease characterized by decreased bone strength and an increased risk of fractures. Osteoporotic fractures, caused by postmenopausal osteoporosis, are the main cause of disability and death in elderly women. These fractures seriously affect the quality of life of patients and bring considerable physical, psychological, and economic burdens to both the patients and their families [1].

Postmenopausal osteoporosis can occur due to many factors. Research has revealed that abnormal hormones, glucose metabolism disorders, and lipid metabolism disorders are associated with bone metabolism disorders and the development of osteoporosis [2–4]. Estrogen is the primary hormone regulating bone metabolism in both women and men [5]. The withdrawal of estrogen is considered the main pathogenesis of PMOP, which induces a marked increase in bone resorption that far outstrips the increase of bone formation by numerous mechanisms, leading to bone mass loss [6–9]. In the past decade, dramatic findings have been achieved regarding the functions of follicle-stimulating hormone (FSH) beyond fertility, especially in bone metabolism [3]. Associations between FSH and bone mass loss had been widely explored. It is generally considered that serum FSH is inversely correlated with BMD, and elevated FSH is positively associated with bone turnover, especially in pre- and perimenopausal women, populations in which estrogen levels remain relatively unchanged [2, 10]. Regarding glucose homeostasis and lipid metabolism, interactions between serum glucose and the skeleton have been revealed in recent years, and diabetes-associated bone disease has come into focus due to the significant detrimental effect that glucose metabolism disorder has on bone [4]. The relationship between dyslipidemia and bone metabolism is still controversial, and some studies have revealed an association between hyperlipidemia and osteoporosis [11–13].

Although the effects of hormones and metabolic markers on bone mass loss in PMOP have been widely studied, conflicting results still exist due to differences in study populations, sample sizes, and covariates adjusted in different researches. Further investigations about the associations between hormones, metabolic marker disorders, and bone mass in PMOP should be conducted. In this research, we studied the relationship between BMD and hormones and metabolic markers in perimenopausal and postmenopausal women. In addition, we explored the risk factors for bone loss in these populations and determined the predictive powers of these risk factors in distinguishing bone loss in the studied population.

## Materials and methods

### Study population

This study was approved by the Ethics Committee of Shandong Provincial Hospital Affiliated to Shandong First Medical University. Written informed consent was obtained from all patients.

A total of 198 perimenopausal and postmenopausal women aged 47–69 years, who received treatment in Shandong Provincial Hospital Affiliated to Shandong First Medical University between January 1, 2021, and December 31, 2022, were enrolled in this study. This included 92 postmenopausal osteoporosis patients, 61 osteopenia patients, and 45 individuals with normal bone mass as controls. The inclusion criteria were as follows: (1) women undergoing menopause or postmenopause, aged 45–70 years; (2) osteoporosis patients who met the diagnostic criteria for osteoporosis (based on a fragility fracture or a dual-energy X-ray absorptiometry assay T-score of − 2.5 or less [14]); (3) those never having suffered from any disease that may affect bone density; (4) those never having received any form of treatment for osteoporosis. The exclusion criteria were as follows: (1) patients suffering from other diseases that affect bone metabolism; (2) patients who are taking or have taken drugs that may affect bone metabolism within the past 6 months, including drugs that affect calcium and phosphorus levels and hormones; (3) patients with subclinical and clinical hypothyroidism/hyperthyroidism; (4) patients with a history of secondary osteoporosis.

All participants had a standardized physical examination including measurement of body weight and height. Body mass index (BMI) was calculated by dividing weight by square of height. Menstrual history, menopause time, and medication history were collected and recorded.

### Bone mineral density measurement

All subjects underwent dual-energy X-ray absorptiometry (Norland, XR-36) examination for the femoral neck and total hip BMD (g/cm^2^). The World Health Organization (WHO) definition was used to diagnose osteoporosis, according to the T-score of femoral neck BMD. Osteoporosis was defined as a T-score ≤ − 2.5; osteopenia as − 2.5 < T-score < − 1; and normal bone mass as T-score ≥ − 1.

### Hormone and metabolic marker detection

Blood samples used for hormone and metabolic marker detection were collected in the morning after an 8–12 h overnight fast. Serum was separated by a 3000 r/min centrifugation for 10 min. Hormones and parts of metabolism indexes including follicle-stimulating hormone (FSH), luteinizing hormone (LH), estradiol (E2), testosterone (T), insulin (INS), c-peptide, prolactin (PRL), parathyroid hormone (PTH), bone-γ-carboxyglutamate protein (BGP), β-carboxy-terminal cross-linked telopeptide of type 1 collagen (β-CTX), 25-hydroxy vitamin D (25-(OH)-D), and procollagen type 1 N-terminal propeptide (P1NP) were detected by Roche Cobas e602 electrochemical immunoassay analyzer and supporting reagents. Metabolic markers and ions were measured by Backman Coulter AU5800 automated biochemical analysis system, including triglyceride (TG, enzyme end (GPO-POD) method), total cholesterol (TC, enzyme end (CHO-POD) method), HDL-cholesterol (HDL-C, selective inhibition method), LDL-cholesterol (LDL-C, surfactant removal method), glucose (GLU, hexokinase end point method), uric acid (UA, uric acid enzyme method), phosphate (phosphomolybdic acid method), calcium (Ca, azo arsine III method), and magnesium (Mg, Xylidyl Blue method). Glycated hemoglobin A1c (HbA1c) was detected by high-pressure liquid chromatography, using TOSOH automated glycohemoglobin analyzer HLC-723G8.

### Statistical analysis

The Shapiro–Wilk normality test was used to assess the distribution of the data. Results are expressed as mean (mean ± SD) for continuous Gaussian variables or median (interquartile range) for continuous non-Gaussian variables. The statistical analysis of the research variables for perimenopausal and postmenopausal women was conducted using the Mann–Whitney *U* test. Kruskal–Wallis one-way ANOVA tests (Gaussian variables) and Kruskal–Wallis *H* tests (non-Gaussian variables) were used to examine differences for all variables between normal bone mass and osteopenia and osteoporosis patients. The comparison of the prevalence of perimenopausal and diabetes was analyzed using the Chi-square test. Spearman correlation analysis and partial correlation analysis were used to analyze correlations of hormones and metabolic markers with BMD. Spearman correlation coefficient (*r*) value greater than 0.2 or less than − 0.2 were considered as significant correlation. Risk factors for bone mass loss were identified by univariate and multivariate logistic regression analysis. To determine the predictive effect of variables for bone mass loss event, the receiver operating characteristic (ROC) curve analysis was performed by measuring the area under the curve (AUC). An AUC > 0.6 was considered acceptable. All analyses were conducted using the Statistical Package for Social Sciences version 27.0.1 (IBM). Graph was plotted using GraphPad Prism version 10.0 (GraphPad). A two-sided *p* < 0.05 was considered to be statistically significant.

## Results

### Characteristics of the participants

**Fig. 1:**
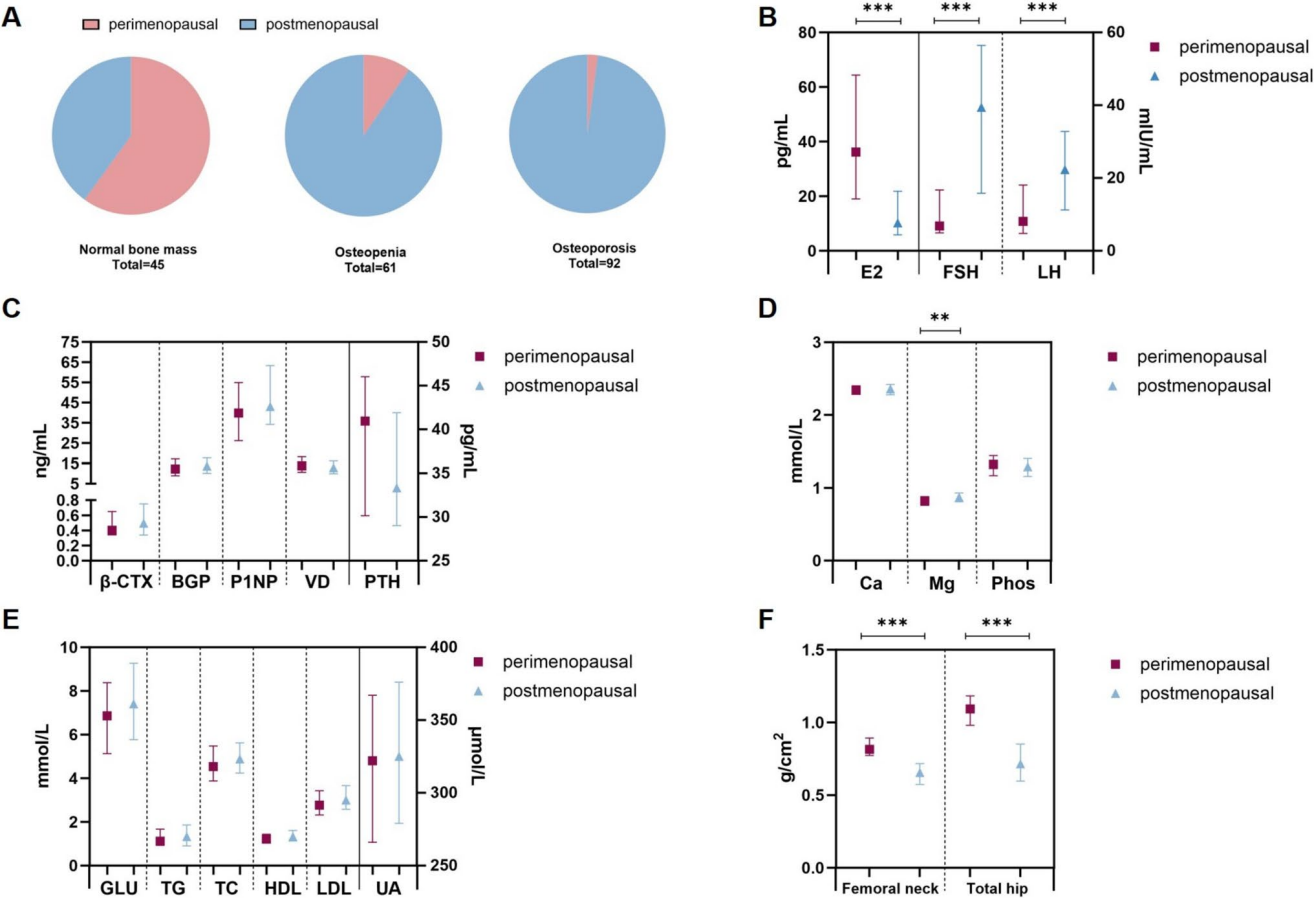
Clinical characteristics of participants and comparison between perimenopausal and postmenopausal women. **a** Proportion of perimenopausal and postmenopausal women in different bone mass groups. **b** Comparison of sex hormone levels in perimenopausal and postmenopausal women. **c **Comparison of bone metabolism parameters. **d** Comparison of ion levels. **e** Comparison of metabolite parameters. **f** Comparison of bone density. Variables were assessed with Mann–Whitney *U* test. Asterisks indicate significant differences between groups (*p < 0.05; **p < 0.01; and ***p < 0.001)

In the study population, 17.68% were perimenopausal women, with 60% belonging to the normal bone mass group, a significantly higher proportion compared to those in the osteopenia and osteoporosis groups. E2 levels in perimenopausal women were notably higher than in postmenopausal women, while FSH and LH levels were significantly lower. Additionally, perimenopausal women exhibited significantly higher bone densities in the femoral neck and total hip regions compared to postmenopausal women. However, no significant differences were observed between perimenopausal and postmenopausal women in other bone metabolism parameters or metabolite parameters (Fig. 1).

Table 1 describes the main characteristics of all studied subjects. Age, years since menopause, and BMI were significantly different between women with normal bone mass and those with osteopenia and osteoporosis. As expected, women with normal bone mass (median age:51, quartile: 47–55) were younger than women with osteopenia (median age: 59, quartile: 53–63) and osteoporosis (median age: 66, quartile: 62–69). BMI was lower in women with osteopenia (25.33 ± 3.99 kg/m^2^) and osteoporosis (23.77 ± 3.56 kg/m^2^) than in women with normal bone mass (26.42 ± 4.08 kg/m^2^). There was no significant difference in the prevalence of diabetes between women with osteopenia (57.38%) and osteoporosis (56.52%). However, the prevalence of diabetes in the normal bone mass group (37.78%) was significantly lower than that in the osteopenia and the osteoporosis group.

**Table 1:** Clinical, hormonal, and metabolic markers’ characteristics of 198 perimenopausal and postmenopausal women according to bone mass status

Variables	Normal bone mass (*N* = 45) T-score > − 1.0	Osteopenia (*N* = 61) T-score (− 2.4 ~ − 1.0)	0steoporosis (*N* = 92) T-score ≤ − 2.5	F/H/P	*p* value*
Age, y	51 (47–55)	59 (53–63)	66 (62–69)	98.336	**< 0.001**
Years since menopause, y	1 (0–5)	8 (4–15)	17 (13–21)	108.15	**< 0.001**
Body mass index, kg/m^2^	26.42 ± 4.08	25.33 ± 3.99	23.77 ± 3.56	7.964	**< 0.001**^¶^
Diabetes	17(37.78%)	35(57.38%)	52(56.52%)	5.09	0.078
PTH, pg/mL	34.41 (28.53–43.12)	33.06 (26.43–43.90)	34.57 (29.63–45.04)	1.2	0.549
BGP, ng/mL	12.32 (9.54–16.80)	12.71 (9.32–16.26)	14.66 (10.66–17.97)	1.85	0.396
β-CTX, ng/mL	0.40 (0.27–0.64)	0.46 (0.34–0.68)	0.54 (0.35–0.84)	6.688	**0.035**
P1NP, ng/mL	39.78 (26.99–53.29)	40.94 (26.77–60.26)	49.46 (38.35–66.13)	11.696	**0.003**
25-(OH)-D, ng/mL	14.20 (11.30–18.17)	12.10 (8.32–17.95)	12.90 (9.93–15.97)	4.509	0.105
TG, mmol/L	1.01 (0.78–1.67)	1.45 (1.02–1.96)	1.23 (0.90–1.83)	6.187	**0.045**
TC, mmol/L	4.37 (3.88–5.47)	4.89 (4.20–5.55)	5.04 (4.40–5.80)	6.684	**0.035**
HDL-C, mmol/L	1.26 (1.08–1.49)	1.25 (1.10–1.48)	1.37 (1.17–1.72)	5.547	0.062
LDL-C, mmol/L	2.62 (2.26–3.41)	2.97 (2.58–3.58)	3.10 (2.61–3.83)	7.633	**0.022**
GLU, mmol/L	6.12 (5.02–7.72)	7.32 (5.85–9.81)	7.75 (6.27–9.37)	14.048	**< 0.001**
UA, μmol/L	310 (247–345)	311 (271–386)	335 (296–385)	5.788	0.055
HbA1c, %	7.4 (6.6–9.6)	8.4 (7.1–10.1)	8.6 (6.6–10.5)	3.599	0.165
Phosphate, mmol/L	1.34 (1.18–1.49)	1.28 (1.13–1.41)	1.29 (1.16–1.40)	3.638	0.162
Calcium, mmol/L	2.37 ± 0.08	2.34 ± 0.15	2.35 ± 0.12	0.652	0.522^¶^
Magnesium, mmol/L	0.84 ± 0.09	0.85 ± 0.09	0.88 ± 0.08	4.091	**0.018**^¶^
INS, μU/mL	7.23 (4.32–15.55)	5.25 (2.91–10.00)	5.35 (2.68–12.65)	5.192	0.075
C-peptide, ng/mL	1.73 (1.12–2.52)	1.69 (0.89–2.35)	1.39 (0.76–2.42)	1.659	0.429
FSH, mIU/mL	6.85 (4.40–25.73)	21.30 (11.10–47.6)	45.87 (28.91–65.09)	55.578	**< 0.001**
LH, mIU/mL	8.06 (4.56–20.71)	18.07 (9.95–26.96)	26.63 (14.96–36.21)	31.351	**< 0.001**
E2, pg/mL	32.92 (15.36–58.83)	16.45 (8.07–31.60)	6.51 (5.31–14.12)	56.844	**< 0.001**
T, ng/mL	0.27 (0.16–0.64)	0.27 (0.13–3.12)	0.19 (0.07–0.33)	16.055	**< 0.001**
Femoral neck (BMD, g/cm^2^)	0.85 (0.79–0.91)	0.69 (0.65–0.79)	0.59 (0.53–0.66)	122.159	**< 0.001**
Total hip (BMD, g/cm^2^)	1.14 (1.08–1.2)	0.81 (0.76–0.90)	0.61 (0.56–0.70)	160.567	**< 0.001**

Values are expressed as mean ± SD or median (interquartile range). Symbol bold in the table denotes statistical significance with a *p* value < 0.05

*Kruskal–Wallis one-way ANOVA tests (Gaussian variables) and ^¶^Kruskal–Wallis *H* tests (non-Gaussian variables) were used to examine differences for variables

### Comparison of hormones and metabolic markers

Bone metabolism indexes β-CTX and P1NP were lower in women with normal bone mass compared with those with osteopenia and osteoporosis. Metabolic markers TG, TC, LDL-C, and GLU were significantly lower in women with normal bone mass than in those with bone mass loss. Mg showed a statistically significant difference between the different groups, but considered to have no clinical significance (Table 1).

For hormone levels, women with normal bone mass exhibited significantly lower concentrations of FSH and LH compared to those with osteopenia and osteoporosis. Conversely, women with normal bone mass demonstrated significantly higher levels of E2 and T relative to those with osteopenia and osteoporosis (Table 1).

### Association of hormones and metabolic markers with BMD

**Fig. 2:**
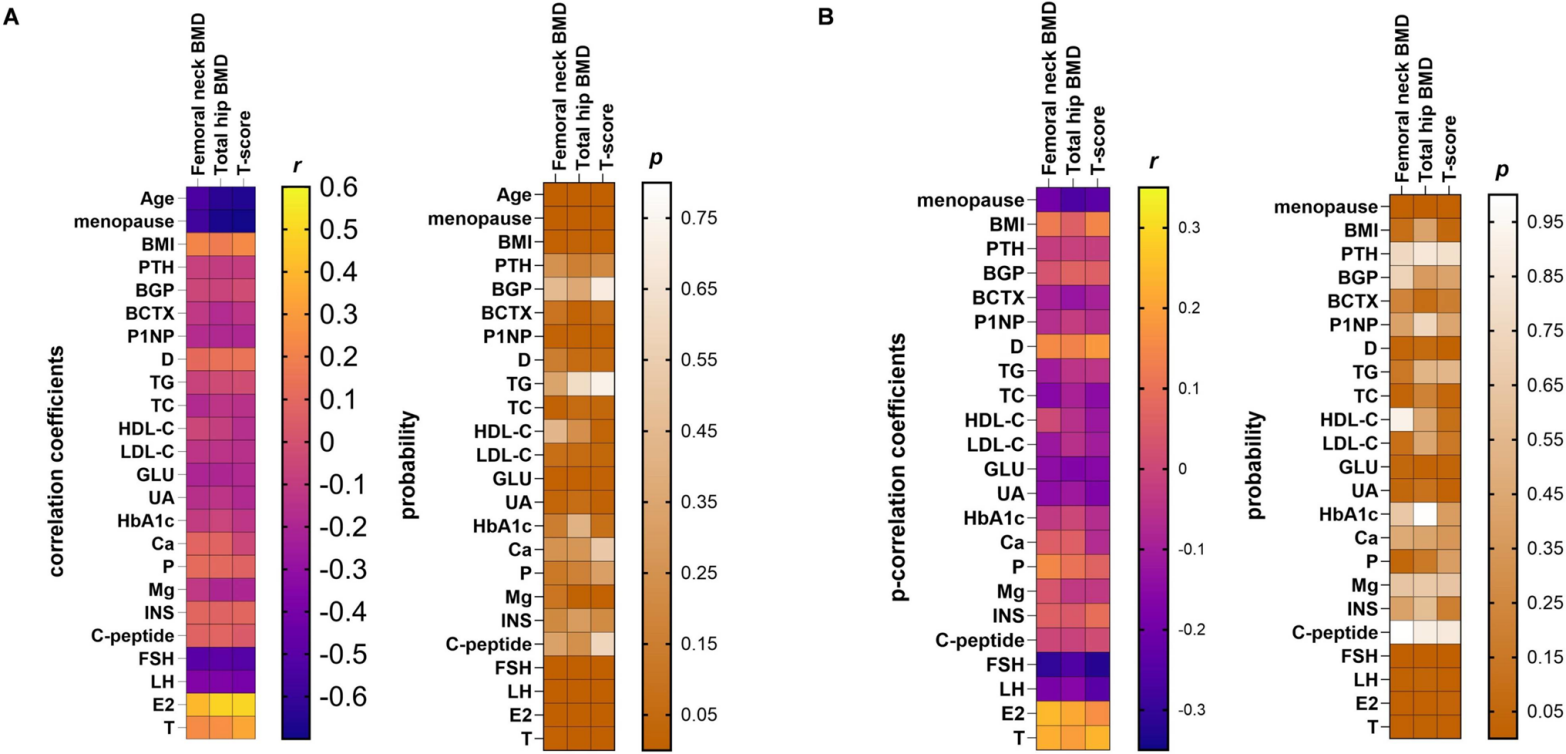
Correlations between hormones and metabolic markers with BMD studied by Spearman correlation analysis and partial correlation analysis. **a** The color map of Spearman correlation coefficients (*r*) and *p* values calculated from Spearman correlation analysis. **b** The color map of partial correlation coefficients (*r*) and *p* values calculated from partial correlation analysis after adjusting for age. Yellow color indicates a positive correlation, while purple color indicates a negative correlation. All the* p* values are color coded based on the scale on the right

Spearman correlation analysis showed that age, years since menopause, GLU, FSH, and LH had significant inverse correlations with femoral neck and total hip BMD. Conversely, BMI, E2, and T had significant positive correlations with femoral neck and total hip BMD (Fig. 2; Table S1). After adjusting for age, the partial correlation analysis revealed that FSH (*r* = − 0.308, *p* < 0.001) had significant negative correlations with femoral neck BMD. E2 (*r* = 0.244, *p* = 0.001) and T (*r* = 0.226, *p* = 0.002) had significant positive correlations with femoral neck BMD. For total hip BMD, the partial correlation analysis showed significant negative correlations with years since menopause (*r* = − 0.259, *p* = 0.006) and FSH (*r* = − 0.257, *p* < 0.001). Additionally, E2 (*r* = 0.215, *p* = 0.003) had a significant positive correlation with total hip BMD (Fig. 2; Table S2).

Since the T-score of BMD has a better evaluation effect on bone loss in patients, we further studied correlations between T-score of femoral neck BMD and hormones and metabolic markers. Spearman correlation analysis displayed significant inverse correlations with age, years since menopause, FSH, and LH. In addition, BMI, E2, and T were significantly positively correlated with the T-score of femoral neck BMD. After adjusting for age, T-score of the femoral neck BMD still had significant negative correlations with years since menopause, FSH, and LH. (Fig. 2; Tables S1 and S2).

### Risk factors for bone mass loss

To explore possible risk factors for bone mass loss in the studied perimenopausal and postmenopausal women, we performed univariate and multivariate binary logistic regression analysis. The occurrence of bone mass loss (osteopenia or osteoporosis) was set up as the dependent variable. Univariate analysis showed that age, years since menopause, the prevalence of diabetes and the levels of β-CTX, P1NP, TC, LDL, GLU, UA, FSH, and LH were risk factors and BMI, 25-(OH)-D, phosphate, E2, and T were protective factors for bone loss in the studied women (Table 2). Then we performed multivariate binary logistic regression analysis including all variables which had significant influence of bone loss identified by univariate logistic regression analysis, except for years since menopause, because there was collinearity between menopausal time and age. This model revealed that age (odds ratio [OR] 1.223; 95% confidence interval [CI] 1.106–1.372; *p* < 0.001), GLU (OR 1.848; 95% CI 1.116–3.059; *p* = 0.017), and FSH (OR 1.089; 95% CI 1.003–1.182; *p* = 0.042) were risk factors for bone mass loss. No significant effect of other variables enrolled in this model was detected (Table 2).

**Table 2:** Univariate and multivariate binary logistic regression analysis to identify risk factors for bone mass loss

Variables	Univariate analysis			Multivariate analysis		
	OR	95% CI	*p*	OR	95% CI	*p*
Age	1.271	1.183–1.364	< 0.001	**1.232**	**1.106–1.372**	**< 0.001**
Menopause	1.444	1.288–1.620	< 0.001			
Diabetes	2.171	1.097–4.295	0.026	0.345	0.056–2.139	0.253
BMI	0.882	0.811–0.959	0.003	0.911	0.795–1.045	0.183
PTH	1.018	0.990–1.046	0.207			
BGP	0.997	0.941–1.056	0.913			
BCTX	4.295	1.160–15.902	0.029	3.452	0.323–36.916	0.305
P1NP	1.023	1.003–1.042	0.022	0.989	0.953–1.027	0.577
25-(OH)-D	0.924	0.869–0.983	0.013	0.916	0.825–1.017	0.101
TG	1.249	0.801–1.946	0.327			
TC	1.341	1.011–1.779	0.041	0.914	0.369–2.259	0.845
HDL	1.434	0.572–3.591	0.442			
LDL	1.621	1.095–2.400	0.016	1.585	0.486–5.172	0.445
GLU	1.381	1.156–1.650	< 0.001	**1.848**	**1.116–3.059**	**0.017**
UA	1.005	1.001–1.009	0.029	1.008	1.000–1.015	0.058
HbA1c	1.161	0.990–1.362	0.067			
Calcium	0.312	0.020–4.850	0.405			
Phosphate	0.131	0.023–0.743	0.022	0.102	0.004–2.795	0.176
Magnesium	46.598	0.804–2700.49	0.064			
INS	0.965	0.927–1.005	0.085			
C-peptide	0.874	0.718–1.064	0.180			
FSH	1.065	1.040–1.091	< 0.001	**1.089**	**1.003–1.182**	**0.042**
LH	1.071	1.036–1.106	< 0.001	0.935	0.848–1.031	0.179
E2	0.950	0.931–0.969	< 0.001	0.988	0.960–1.017	0.414
T	0.833	0.698–0.994	0.043	1.239	0.899–1.707	0.191

### Bone mass loss prediction model

**Fig. 3:**
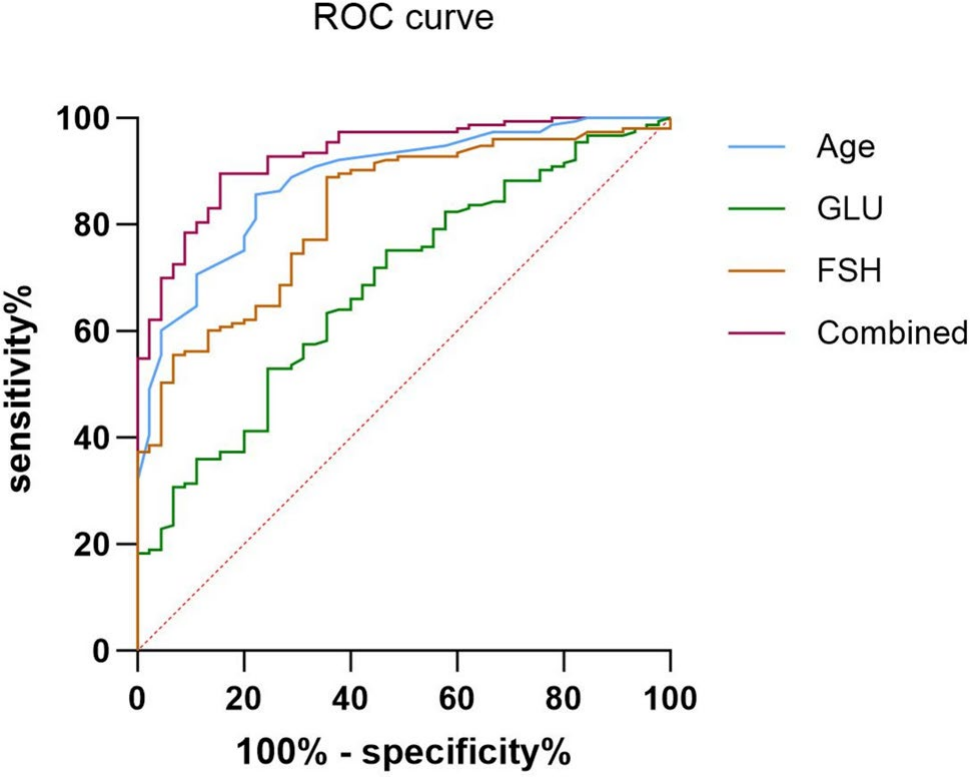
ROC curves for predicting bone mass loss by using age and levels of FSH and GLU, either independently or in combination. The vertical axis represents sensitivity, and the horizontal axis represents 1-specificity

Using ROC curve analysis, the optimal cutoff values of risk factors in the prediction of bone mass loss (osteopenia or osteoporosis) occurrence were identified (Fig. 3; Table S3). Age (AUC = 0.884, 95% CI 0.833–0.935) and FSH (AUC = 0.824, 95% CI 0.760–0.888) exhibited significant predictive power for bone mass loss, and the optimal cutoff value for age was 55 years (sensitivity 85.6%, specificity 77.8%) and for FSH 9.26 mIU/mL (sensitivity 88.9%, specificity 64.4%). GLU (AUC = 0.683, 95% CI 0.599–0.768) showed moderate discrimination capability of bone mass loss, and the optimal cutoff point was 6.14 mmol/L (sensitivity 75.2%, specificity 53.3%).

To evaluate the predictive value of the combined use of these three risk factors for bone mass loss, we constructed a multivariate logistic regression model including age, GLU, and FSH and obtained a regression formula as follows: ln(*p*/1 − *p*) = − 14.773 + 0.212 * age + 0.401 * glu + 0.042 * FSH, where *p* represents the probability of bone mass loss. Hosmer–Lemeshow test showed a good fit of this model (χ^2^ = 3.272, *p* = 0.916), Table S4. The combined use of all three risk factors by multivariate logistic regression showed a significant prediction power (AUC = 0.930, 95% CI 0.893–0.967) for bone mass loss in perimenopausal and postmenopausal women. A probability of 0.695 (sensitivity 89.5%, specificity 84.4%) was considered to be the optimal cutoff point for this prediction model (Fig. 3; Table S3).

## Discussion

In this study, we analyzed the correlations between hormones, metabolic markers, and BMD in perimenopausal and postmenopausal women, discussing the effects of these hormones and metabolic markers on bone mass loss in the studied population. Hormone metabolism disorders, which naturally follow the aging process, are primary factors in postmenopausal osteoporosis, especially the decline of estrogen. Estrogen is an important regulator of bone metabolism that is able to inhibit osteoclast differentiation and promote osteoclast apoptosis [6]. Estrogen deficiency partially relieves its inhibitory effect on osteoclast differentiation, resulting in increased osteoclast activation, which is the fundamental pathological change in postmenopausal osteoporosis [7–9, 15, 16]. Androgen also plays important roles in bone metabolism, both through direct actions on the bone in the form of testosterone and indirect actions by transforming into estradiol [5]. The decline in androgen is commonly considered a secondary cause of osteoporosis in elderly men [17]. Despite the significant roles and mechanisms revealed about estrogen deficiency in PMOP, many studies have supported an estrogen-independent role of FSH in bone metabolism in recent decades [18, 19]. Most studies suggest that high serum FSH is inversely correlated with BMD in women; however, there are also some conflicting pieces of evidence showing that no associations exist between FSH and BMD [20, 21].

E2 and FSH levels exhibited significant differences between perimenopausal and postmenopausal women in our studied population, while E2 and FSH levels were significantly different between women with normal bone mass and those with bone loss (osteopenia and osteoporosis). These findings are mainly consistent with the established understanding that sex hormones change with increasing age [22]. Estrogen deficiency has long been considered a significant factor in postmenopausal bone loss. However, during the early stages of menopause, estrogen levels remain relatively stable, whereas FSH levels increase markedly [23]. The Study of Women’s Health Across the Nation (SWAN) found that in premenopausal and perimenopausal women, serum FSH is significantly negatively correlated with BMD, while estrogen has no significant correlation with BMD, emphasizing the independent risk role of high FSH in early menopausal transition bone loss [10]. In our study, we found that after menopause, women’s E2 decreased, while FSH increased, and E2 was significantly positively correlated, while FSH was significantly negatively correlated with both femoral neck and total hip BMD. In addition, our analysis revealed a significant positive correlation between FSH and both β-CTX and PINP (data not shown) levels, suggesting that elevated FSH levels are associated with increased bone turnover. Univariate regression analysis confirmed that low E2 and high FSH are independent risk factors for bone loss after entering menopause. Multivariate regression analysis showed that estrogen had no effect on bone loss, which may be due to some variables included being confounders for this hormone, especially aging. However, FSH was still significantly associated with increased risk of bone mass loss, which indicated elevated FSH as a confirmed risk factor for bone loss.

The prevalence of diabetes is significantly lower in populations with normal bone mass than in those with osteopenia and osteoporosis. Numerous studies have established that diabetes mellitus is a significant risk factor for osteoporosis, with elevated blood glucose and adipokines exerting detrimental effects on the skeletal system [24]. Generally, it is believed that the risk of fractures increases in patients with diabetes, but the mechanisms underlying this increased risk may vary between different types of diabetes. In terms of bone mineral density, patients with type 1 diabetes have lower BMD compared to non-diabetic individuals, while those with type 2 diabetes have higher BMD [25]. In patients with type 2 diabetes, poor blood glucose control may be a significant factor contributing to bone damage [25]. Elevated blood glucose can impair osteoblast function by inducing resistance to IGF-1 in osteoblasts and by increasing the damage to osteoblasts' responsiveness to IGF-1 through advanced glycation end products [26, 27]. This finding suggests that the increased fracture risk in patients with type 2 diabetes may be related to alterations in bone microarchitecture, rather than a decrease in bone density. In our study population, the femoral neck bone density of diabetic patients was marginally lower compared to non-diabetic patients, while no significant difference in total hip bone density was observed between the two groups (data not shown). Univariate regression analysis indicated that diabetes may be a potential risk factor for bone loss. However, due to limited clinical data, we were unable to classify the types of diabetes within our study population. Consequently, it is challenging to elucidate the relationship between different types of diabetes and bone density in perimenopausal and postmenopausal populations based on this study. Future research should focus on improving the classification of diabetes types and increasing the sample size to better understand the correlation between specific types of diabetes and bone loss.

The roles of metabolic markers including serum glucose and lipid in bone metabolism had been widely reported [4]. We observed significant increases in the levels of GLU, TG, TC, and LDL-C in women with osteopenia and osteoporosis than in those with normal bone mass. The prevalence of diabetes is significantly lower in populations with normal bone mass than in those with osteopenia and osteoporosis. Indeed, elevated glucose levels activate the non-canonical Wnt/protein kinase C pathway and upregulate peroxisome proliferator-activated receptor gamma (PPARγ), resulting in enhanced adipogenesis and bone loss [28, 29]. Consequently, inadequate glycemic control in diabetic patients may suppress key genes critical for osteoblastogenesis, such as Runx2 [30]. Moreover, in vitro studies indicate that high glucose concentrations and advanced glycation end products (AGEs) elevate the expression of sclerostin in osteocytes, a negative regulator of bone formation. Clinical investigations have corroborated these preclinical findings, demonstrating that sclerostin levels are significantly higher in prediabetic individuals compared to controls and are associated with insulin resistance [31]. Collectively, these studies highlight the adverse effects of hyperglycemia on bone metabolism. Nonetheless, further research is warranted to fully elucidate the relationship between blood glucose levels and osteoporosis. In our study, we confirm that GLU negatively correlated with femoral neck and total hip BMD. After adjusting for age, the correlation coefficients decreased but were still significant, which suggest that BMD in the studied perimenopausal and postmenopausal women decreased with serum GLU, consistent with previous reports in type 1 diabetes mellitus (T1DM) [32, 33]. Univariate and multivariate logistic regression analysis both showed that higher serum GLU was a risk factor for bone loss in our study. However, a large cohort study in Korean, which aimed to evaluate the risk of incident osteoporosis according to fasting glucose, revealed a protective effect of elevated fasting glucose on osteoporosis [34]. These conflicting results are a reminder that we should be careful when judging the effect of serum glucose on osteoporosis.

The major strength of our study lies in the analysis of the effectiveness of using multiple indicators in combination to assess bone loss. Since the use of a single indicator for assessing the risk of bone loss is often limited, and the concurrent utilization of multiple indicators warrants greater attention. ROC curve analysis shows that the variables affecting bone loss obtained from regression analysis, such as age, FSH, and GLU, have good diagnostic efficacy in predicting bone loss, and their combined use can significantly improve predictive ability. Therefore, monitoring FSH and GLU levels in women after menopause can help assess their risk of bone loss. For older women with high levels of FSH and GLU, increasing the frequency of bone density monitoring can enhance the ability to prevent and diagnose osteoporosis early.

Nonetheless, limitations of our study should be documented. Firstly, the sample size of women with normal bone mass was relatively small (*N* = 45), which may introduce bias into the regression analysis. To mitigate this potential bias, we initially conducted univariate regression analyses and subsequently included only those variables that demonstrated a significant effect on bone loss in the final multivariate regression model, thereby controlling the number of variables entering the regression analysis. Secondly, our dataset lacks information on lifestyle factors such as smoking, alcohol consumption, and family history, including parental fragility fractures. These variables are potential confounders that could influence the associations between the hormones and metabolic markers we studied and osteoporosis. Consequently, residual confounding may be present in our study. Despite these limitations, all research data were collected using standardized experimental methods, ensuring the accuracy and reliability of the measurements and providing a true reflection of the actual levels of these markers in the study population. This approach effectively captures the relationships between hormones, metabolic indicators, and postmenopausal osteoporosis.

## Conclusion

In conclusion, our study revealed a negative correlation between follicle-stimulating hormone (FSH) and glucose (GLU) levels with bone mineral density (BMD) in perimenopausal and postmenopausal women. We found that aging and elevated FSH and GLU levels were associated with an increased risk of bone loss. The independent or combined use of these factors provides substantial predictive power for bone mass loss in the studied population.

## Supplementary Information

Below is the link to the electronic supplementary material.

Supplementary file 1 (DOCX 26 KB)

## Data Availability

The authors confirm that the data supporting the findings of this study are available within the article and its supplementary materials.

## Abbreviations

ROC: Receiver operating characteristic
GLU: Glucose
FSH: Follicle-stimulating hormone
LH: Luteinizing hormone
OR: Odds ratio
CI: Confidence interval
PMOP: Postmenopausal osteoporosis
WHO: World Health Organization
BGP: Bone-γ-carboxyglutamate protein
PRL: C-peptide, prolactin
PTH: Parathyroid hormone
β-CTX: β-Carboxy-terminal cross-linked telopeptide of type 1 collagen
T1DM: Type 1 diabetes mellitus
IOF: International Osteoporosis Foundation
IFCC: International Federation of Clinical Chemistry and Laboratory Medicine
FSH: Follicle-stimulating hormone
TC: Total cholesterol
UA: Uric acid
BMD: Bone mineral density
