# Supplementary material for: Associations between hormones, metabolic markers, and bone mass in perimenopausal and postmenopausal women
Source: J Bone Miner Metab. 2025 Mar 5;43(4):392–401. doi: 10.1007/s00774-025-01595-x (PMC12279895; doi:10.1007/s00774-025-01595-x)
Supplement: Supplementary file 1 — (DOCX 26 KB) [file 774_2025_1595_MOESM1_ESM.docx]

Supplement Table1: Analysis of the correlation between hormones and metabolic markers with BMD by Spearman correlation analysis.

Spearman correlation coefficients (r) and p values were shown.

| Variables | Femoral neck BMD | | Total hip BMD | | T-score | |
| --- | --- | --- | --- | --- | --- | --- |
|  | *r* | *p* | *r* | *p* | *r* | *p* |
| Age | **-0.539** | **<0.001** | **-0.641** | **<0.001** | **-0.658** | **<0.001** |
| Years since menopause | **-0.574** | **<0.001** | **-0.679** | **<0.001** | **-0.691** | **<0.001** |
| Body mass index | **0.213** | **0.003** | **0.174** | **0.014** | **0.235** | **0.001** |
| PTH | -0.082 | 0.249 | -0.101 | 0.157 | -0.09 | 0.206 |
| BGP | -0.054 | 0.451 | -0.065 | 0.362 | -0.027 | 0.703 |
| β-CTX | -0.118 | 0.099 | **-0.176** | **0.013** | -0.133 | 0.063 |
| PINP | **-0.17** | **0.017** | **-0.19** | **0.007** | **-0.195** | **0.006** |
| 25-(OH)-D | 0.103 | 0.148 | 0.136 | 0.056 | **0.144** | **0.043** |
| TG | -0.068 | 0.339 | -0.035 | 0.621 | -0.025 | 0.728 |
| TC | **-0.18** | **0.011** | -0.134 | 0.059 | **-0.149** | **0.036** |
| HDL-C | -0.056 | 0.436 | -0.085 | 0.235 | **-0.164** | **0.021** |
| LDL-C | -0.127 | 0.075 | -0.135 | 0.059 | **-0.151** | **0.033** |
| GLU | **-0.205** | **0.004** | **-0.2** | **0.005** | **-0.181** | **0.011** |
| UA | **-0.157** | **0.028** | -0.131 | 0.066 | **-0.183** | **0.01** |
| HbA1c | -0.103 | 0.148 | -0.059 | 0.41 | -0.125 | 0.079 |
| Phosphate | 0.081 | 0.254 | 0.079 | 0.268 | -0.046 | 0.523 |
| Calcium | 0.11 | 0.122 | 0.098 | 0.171 | 0.072 | 0.314 |
| Magnesium | -0.117 | 0.102 | **-0.196** | **0.006** | **-0.2** | **0.005** |
| INS | 0.09 | 0.207 | 0.074 | 0.301 | 0.088 | 0.216 |
| C-peptide | 0.071 | 0.324 | 0.084 | 0.241 | 0.039 | 0.586 |
| FSH | **-0.497** | **<0.001** | **-0.484** | **<0.001** | **-0.513** | **<0.001** |
| LH | **-0.366** | **<0.001** | **-0.373** | **<0.001** | **-0.404** | **<0.001** |
| E2 | **0.401** | **<0.001** | **0.487** | **<0.001** | **0.492** | **<0.001** |
| T | **0.243** | **0.001** | **0.257** | **<0.001** | **0.336** | **<0.001** |

Supplement Table2: correlations between hormones and metabolic markers with BMD by partial correlation analysis after adjusting for age.

partial correlation coefficients (*p-r*) and p value were shown.

| Variables | Femoral neck BMD | | Total hip BMD | | T-score | |
| --- | --- | --- | --- | --- | --- | --- |
|  | *p-r* | *p* | *p-r* | *p* | *p-r* | *p* |
| Years since menopause | **-0.197** | **0.006** | **-0.259** | **<0.001** | **-0.237** | **0.001** |
| Body mass index | 0.121 | 0.094 | 0.059 | 0.417 | **0.144** | **0.046** |
| PTH | -0.022 | 0.760 | -0.014 | 0.849 | -0.018 | 0.801 |
| BGP | 0.027 | 0.714 | 0.067 | 0.357 | 0.058 | 0.419 |
| β-CTX | -0.088 | 0.226 | -0.127 | 0.077 | -0.094 | 0.192 |
| PINP | -0.060 | 0.405 | -0.024 | 0.740 | -0.057 | 0.433 |
| 25-(OH)-D | **0.153** | **0.033** | 0.137 | 0.057 | **0.182** | **0.011** |
| TG | -0.105 | 0.145 | -0.046 | 0.529 | -0.045 | 0.537 |
| TC | **-0.156** | **0.031** | -0.091 | 0.209 | **-0.149** | **0.039** |
| HDL-C | 0.008 | 0.907 | -0.056 | 0.440 | -0.119 | 0.100 |
| LDL-C | -0.119 | 0.098 | -0.056 | 0.436 | -0.104 | 0.149 |
| GLU | **-0.142** | **0.049** | **-0.165** | **0.021** | **-0.156** | **0.030** |
| UA | **-0.143** | **0.048** | -0.114 | 0.113 | **-0.168** | **0.019** |
| HbA1c | -0.033 | 0.646 | 0.001 | 0.990 | -0.063 | 0.383 |
| Phosphate | 0.054 | 0.458 | 0.058 | 0.424 | -0.068 | 0.345 |
| Calcium | **0.146** | **0.042** | 0.104 | 0.152 | 0.062 | 0.388 |
| Magnesium | 0.035 | 0.633 | -0.032 | 0.655 | -0.034 | 0.634 |
| INS | 0.060 | 0.407 | 0.040 | 0.583 | 0.094 | 0.193 |
| C-peptide | -0.001 | 0.994 | -0.010 | 0.890 | 0.014 | 0.851 |
| FSH | **-0.308** | **<0.001** | **-0.257** | **<0.001** | **-0.322** | **<0.001** |
| LH | **-0.186** | **0.010** | **-0.159** | **0.027** | **-0.240** | **0.001** |
| E2 | **0.244** | **0.001** | **0.215** | **0.003** | **0.162** | **0.025** |
| T | **0.226** | **0.002** | **0.193** | **0.007** | **0.236** | **0.001** |

Supplement Table3: The predictive power of age, FSH, GLU and the combined use of these three factors in bone loss, as demonstrated through ROC curve analysis.

| Variable | AUC | 95%CI | *P* value | Youden | Optimal cutoff value | Sensitivity | Specificity |
| --- | --- | --- | --- | --- | --- | --- | --- |
| Age, y | 0.884 | [0.833-0.935] | <0.001 | 0.634 | 55 | 0.856 | 0.778 |
| GLU, mmol/L | 0.683 | [0.599-0.768] | <0.001 | 0.285 | 6.14 | 0.752 | 0.533 |
| FSH, mIU/mL | 0.824 | [0.760-0.888] | <0.001 | 0.533 | 9.26 | 0.889 | 0.644 |
| Combined,（*p*） | 0.930 | [0.893-0.967] | <0.001 | 0.740 | 0.695 | 0.895 | 0.844 |

Supplement Table4: Multivariate logistic regression analysis enrolled age, GLU and FSH as independent variables for bone mass loss.

| Variables | β | Strand error | Wald | P value | odds ratio | OR 95%CI |
| --- | --- | --- | --- | --- | --- | --- |
| Age | 0.212 | 0.041 | 26.522 | <0.001 | 1.237 | 1.117-1.354 |
| GLU | 0.401 | 0.121 | 11.068 | 0.001 | 1.494 | 1.160-2.041 |
| FSH | 0.042 | 0.014 | 9.032 | 0.003 | 1.043 | 1.015-1.187 |
| Constant | -14.773 | 2.568 | 33.088 | <0.001 | <0.001 | / |
